# The Fluctuating Natural Course of CCSVI in MS Patients and Controls, a Prospective Follow-Up

**DOI:** 10.1371/journal.pone.0078166

**Published:** 2013-11-19

**Authors:** Petronella J. Van den Berg, Leo H. Visser

**Affiliations:** Department of Neurology, St. Elisabeth Hospital, Tilburg, The Netherlands; University Hospital La Paz, Spain

## Abstract

**Objectives:**

A new treatable venous disorder, chronic cerebrospinal venous insufficiency (CCSVI), has been proposed in patients with multiple sclerosis. The natural course of CCSVI has not been examined yet. This is crucial given the fact that surgical procedures are increasingly offered to MS patients to treat venous stenosis.

**Methods:**

To document the natural course of venous haemodynamics we performed extra- and transcranial echo colour Doppler (ECD) in 52 multiple sclerosis patients and 28 healthy controls (HC) and re-examined this group after a median period of 16 weeks. The reexamination was done being blinded to the initial findings and the patients did not undergo any intervention.

**Results:**

The ECD examination at baseline showed CCSVI in 5 (9.6%) of the 52 multiple sclerosis patients and 0 HC (P = 0.26). At follow-up the diagnosis CCSVI could not be reconfirmed in 3 out of 5 patients at follow-up, while 2 new CCSVI-positive multiple sclerosis patients were detected.

**Conclusions:**

ECD examination shows a fluctuating natural course of the extracranial venous haemodynamics, which makes determination of CCSVI by ECD examination unreliable.

## Introduction

A new treatable venous disorder, chronic cerebrospinal venous insufficiency (CCSVI), as proposed by Zamboni et al. [1], [2] is a vascular condition characterized by an impaired cerebrospinal venous drainage due to obstructions in the main extracranial cerebrovenous outflow routes (i.e. internal jugular veins (IJV) and/or azygos veins) and causing iron accumulation outside the veins in the central nervous system. CCSVI has been suggested to have a parallel to chronic venous disorders in lower extremities with abnormal iron deposition around the veins [3], [4].

CCSVI in MS has received enormous attention among patients, physicians and media [5], because of the possible new pathogenetic insights of MS and the potentially treatment possibilities [6], [7] which already have resulted in an as yet, unproven treatment with its possible risks. The aim of our study was to examine the natural course of the extracranial venous dynamics, which, to our knowledge, has never been documented before.

## Methods

### Participants

This single centre, prospective, rater-blinded study included a subgroup of 52 patients with MS and 28 healthy age- and sex-matched subjects who participated in our recent published study [8]. The patients and controls were selected at random without knowing the initial transcranial and extracranial venous echo color Doppler (ECD) results. The study was approved by the local medical ethical committee of the St Elisabeth hospital, Tilburg (CCMO number: NL33659.008.10) and informed consent was obtained from each subject prior to the investigation. All participants provided their written informed consent to participate in this study.

#### Clinical characteristics and assessments

Inclusion criteria were age between 18 and 65 years and definite MS, diagnosed according to the revised McDonald diagnostic criteria [9].

The exclusion criteria for both groups were: previous history of cerebral venous thrombosis or vascular malformations, previous central venous catheter in the IJV, previous head and neck surgery and a relapse or steroid treatment within the 30 days prior to study entry. All patients underwent a clinical neurological assessment with determination of the expanded disability status scale (EDSS) score [9].

### Echo colour Doppler study of cerebral venous return

All subjects were examined using transcranial and extracranial venous ECD for studying the deep cerebral, internal jugular and vertebral veins (VV) [1], [2]. The methods of this technique have been described extensively in our manuscript describing the occurrence of CCSVI in the Dutch MS population [8].

The following five criteria were assessed as described in our recent paper [10]:

1. Reflux in the IJVs and/or VVs in supine and upright position (criterion I)
2. Reflux in the deep cerebral veins (DCVs) (criterion II)
3. High resolution B-mode proximal IJV stenosis (criterion III)

High-resolution B-mode evidence of IJV stenosis was assessed in both transversal and longitudinal plane in both colour and gray scale in a recumbent position. Two techniques are used:

- a IJVs stenosis as a local CSA reduction of ≥50% across the IJV (method 1), and
- a cross sectional area (CSA) of the IJV of ≤0.3 cm^2^ measured at the level of the thyroid gland (method 2), or determining a stenosis as only B-mode anomalies.

1. Flow not Doppler detectable in IJVs and/or VVs (criterion IV)
2. Reverted postural control of the main cerebrovenous outflow pathway (criterion V). The delta (Δ) CSA was calculated by subtracting the upright from the supine CSA (ΔCSA  =  CSA_supine_ – CSA_upright_).

#### Assessment of CCSVI criteria

The presence of ≥2 criteria was considered CCSVI-positive.

### Natural course of extracranial venous haemodynamics

In all MS patients and HC a follow-up ECD was performed to assess the natural course of the cerebrovenous dynamics. The participants did not undergo any mechanical or medical intervention in the meantime. All study examinations of the first ECD assessment and follow-up ECD were performed and interpreted by the same 3 ECD technologists. They were fully blinded to the first ECD data, the subjects diagnostic category and unaware of how many subjects per category were enrolled in the study. At follow-up, the entire ECD as performed at baseline was repeated. At baseline and at follow-up, both method 1 and 2 for stenosis measurements (criterion III) were used. There was one change in the protocol, based on the the results of the first DCVs (n = 130) [8]. Because we did not detect any reflux (criterion II), we decided not to repeat this examination at the second assessment.

To evaluate the physiological variations of the internal jugular vein diameter [11], we prospectively evaluated the IJV CSA at both sides in one HC 21 times during a follow-up period of 4 months with insonation at an identical site, e.g. at the level of the thyroid gland in transverse plane.

### Statistical analysis

Data were analysed using MedcalcVersion 11. For descriptive statistic and estimates of prevalence, t tests, Fisher exact tests (two-sided), and X2 tests were used to evaluate statistically significant relationships between categorical variables. P-values less than 0.05 were considered statistically significant.

## Results

### Participant characteristics

A total of 52 MS patients (11 men) and 28 HC (14 men) were included in this study. Demographics of both groups are presented in Table 1. Secondary progressive (SP) and primary progressive (PP) patients were older and had more neurological disability in comparison to RR patients. Disease duration was longest in SP-MS patients.

**Table 1:** Demographics of 52 patients with MS and 28 controls.

Clinical	All MS patients	RR-MS	SP-MS	PP-MS	Controls
Characteristics	n = 52 (%)	n = 35 (67.3)	n = 9 (17.3)	n = 8 (15.4)	n = 28
Age, y	49.0	47.0	52.0	52.0	42.0
median (SD)	(9.6)	(10.0)	(6.4)	(6.4)	(11.4)
Sex %M	21.2%	20.0%	22.2%	25.0%	50.0%
(M/F)	(11/41)	(7/28)	(2/7)	(2/6)	(14/14)
Disease duration, months,	84.0	60.0	180.0	47.0	NA
median (95% CI)	(43.8–138.4)	(35.2–129.1)	(95.6–232.4)	(16.1–135.9)	
EDSS median (95% CI)	3.5	2.0	6.5	6.0	NA
	(2.2–5.5)	(1.0–3.3)	(6.0–6.5)	(3.4–6.5)	

Abbreviations.

CI, confidence interval; SD, standard deviation; F, female; M, man; EDSS, expanded disability status scale; MS, multiple sclerosis; PP-MS, primary progressive MS; RR-MS, relapsing-remitting MS; SP-MS, secondary progressive MS.

### Natural course of extracranial venous haemodynamics

A second ECD was performed after a median period of 16±5.5 weeks without knowing the initial ECD results. Table 2 reports the findings of the ECD of the five CCSVI criteria in all participants at baseline and at follow-up.

**Table 2:** Natural course of extracranial venous haemodynamics in 52 patients with MS over a median time period of 16

		Baseline	Follow-up
Criterion	Ultrasound n = 52 (%)/N	MS patients n = 52 (%)/N	MS patients n = 52 (%)/N
**I**	Reflux IJVs/VVs	3 (5.8)/52	1 (2.0)/52
	(IJV/VV)	(1/2)	(1/0)
**II**	Reflux DCVs	0 (0.0)/52	NA
**III**	IJVs stenosis		
Method 1	*as a local CSA reduction of ≥50%*	6 (11.5)/52	3 (5.8)/52
Method 2	*as a ≤0.3 cm^2^ at level of the thyroid gland*	5 (9.6)/52	13 (25.0)/52
Methods 1 & 2	*including B-mode anomalies*	18 (34.6)/52	23 (44.2)/52
B-mode anomalies	*IJV stenosis only as B-mode anomalies*	12 (23.1)/52	10 (19.2)/52
**IV**	Absent flow IJVs/VVs	1 (1.9)/52	2 (3.8)/52
	(IJV/VV)	(0/1)	(1/1)
**V**	Negative IJVs ΔCSA	4 (20.0)/52	3 (15.0)/20

At baseline and at follow-up, for stenosis measurements, method 1 and 2 were used.

Method 1 IJVs stenosis as a local CSA reduction of ≥50% across the IJV. Method 2 IJVs stenosis as a ≤0.3 cm^2^ CSA, measured at the level of the thyroid gland.

B-mode anomalies, referring to septum, flap, annulus, web or malformed valve in the lumen of the IJV, indicating stenosis.

ΔCSA  =  CSA_supine_ – CSA_upright_.

Abbreviations

CCSVI, chronic cerebrospinal venous insufficiency; CSA, cross section area; DCV, deep cerebral vein; ECD, echo color Doppler; IJV, internal jugular vein; MS, multiple sclerosis; VV, vertebral vein.

#### Criterion I

During the first assessment we found IJV reflux in both positions and VV reflux in one position (monolateral) in one patient, which appeared unchanged at follow-up. Two patients had reflux in the VVs at baseline, while at follow-up a normal flow was detected.

#### Criterion II

In 8 participants (6 patients) with a local CSA reduction of ≥50% across the IJV (method 1) this reduction was persistent at follow-up in all but one participant. At follow-up, 2 additional patients had a local CSA reduction of ≥50%, while this was not present at baseline. Sixteen participants (13 patients) were documented with a IJV CSA ≤0.3 cm^2^ at the level of the thyroid gland (method 2); this was persistent in only 3 patients at the same side. In one patient, who had this stenosis at both sides, at follow-up this stenosis persisted only at one side. One HC had a local stenosis at the left side and at follow-up the stenosis was present at the other side. In both sessions the right median CSA measured at the level of the thyroid gland was 0.85 cm^2^ (n = 80 (52 patients with MS and 28 HC); SD ±0.47 cm^2^ at baseline and ±0.51 cm^2^ at follow-up, respectively).

A total of 19 participants (12 patients) had B-mode anomalies at baseline. Follow-up ECD redetected 17 of them; one septum and malformed valve could not be redetected. One “malformed” valve, initially resulting into a ≥50% stenosis, functioned normally at follow-up.

#### Criterion III

At baseline 2 participants (1 patient) were documented with a block in the VVs but only the patients block remained unchanged. In the HC at follow-up absence of flow was only detected in the same VVs in the extravertebral to subclavian vein and normal flow was detected from the C3 to C6 level in both po sitions. At follow-up a block was detected in the IJV at follow-up in one patient who initially had a normal flow. At baseline two participants (1 patient) showed absence of flow in just one position in the VVs, at follow-up normal flow was detected in all veins in both positions.

#### Criterion IV

A negative ΔCSA was detected in 5 participants (4 patients) at baseline (n = 25) and this was persistent except for one patient. Due to lack of assessment, criterion V was assessed in 20 patients with MS and 5 HC at follow-up.

#### Prospective evaluation of the internal jugular vein diameter during multiple measurements in one healthy control during a follow-up period of 4 months

Over a median period of 4 months we evaluated during 21 times the CSA of the IJV at both sides at the same level in one HC. In 9 assessments a stenosis (CSA being ≤0.3 cm^2^) was observed in the left IJV, during the other 12 measurements the CSA was normal being ≥0.3 cm^2^. In the right IJV the CSA was normal during all measurements, being ≥0.3 cm^2^.

In the left IJV the CSA had a range of 0.11 cm^2^−0.62 cm^2^ (median 0.35 cm^2^; SD 0.15) in supine position, and 0.05 cm^2^−0.24 cm^2^ in sitting position (range ΔCSA 0.05cm^2^−0.08 cm^2^). In the right IJV the CSA varied from 0.95 cm^2^ to 2.68 cm^2^ (median 1.88 cm^2^; SD 0.49) in supine position, and 0.08 cm^2^−1.50 cm^2^ in sitting position (range ΔCSA 0.35 cm^2^−2.85 cm^2^).

#### Persistence of CCSVI at follow-up

The natural course of CCSVI in 7 patients with MS (5 initially and 2 at follow-up) is presented in Table 3. Only in 2 of the 5 MS patients with CCSVI at baseline we could reconfirm the diagnosis of CCSVI at follow-up ECD (Table 3, patient 1 and patient 3); the assessments being performed 10 and 12 weeks after the initial diagnosis. In one MS patient with CCSVI at baseline, the negative ΔCSA became positive at follow-up assessed 12 weeks after baseline (1^st^ time −0.16 cm^2^; 2^nd^ time +0.61 cm^2^) and the diagnosis CCSVI could not be reconfirmed (Table 3, patient 5). In the other 2 MS patients with the diagnosis CCSVI at baseline (Table 3, patient 2 and patient 4; both 2 criteria; reflux in the VVs and stenosis in the IJVs), the reflux was not present at follow-up ECD, thereby no longer fulfilling the criteria of CCSVI. Due to lack of assessment at follow-up, in both patients criterion V was not assessed.

**Table 3:** Natural course of CCSVI criteria in 7 MS patients with ≥2 criteria over a median time period of 12 weeks.

	Baseline	Follow-up
Participants	Ultrasound CCSVI criteria	Ultrasound CCSVI criteria
(period between sessions in weeks)		
1 (12)	block in VVs (L)	block in VVs (L)
	negative IJVs ΔCSA (L)	negative IJVs ΔCSA (L)
2 (10)	reflux in VVs (L)	NP
	stenosis; a local CSA reduction of ≥50% across IJV (R)	NP
3 (10)	stenosis: a local CSA reduction of ≥50% across IJV (R)	NP
	stenosis: a ≤0.3cm^2^ at thyroid gland (both sides)	stenosis: a ≤0.3 cm^2^ at thyroid gland (both sides)
	negative IJVs ΔCSA (L)	negative IJVs ΔCSA (L)
4 (21)	reflux in VVs (R)	NP
	stenosis: malformed valve (R)	stenosis: malformed valve (R)
	stenosis: a ≤0.3 cm^2^ at thyroid gland (L)	NP
5 (23)	stenosis: a local CSA reduction of ≥50% across IJV (L)	stenosis: a local CSA reduction of ≥50% across IJV (L)
	negative IJVs ΔCSA (L)	NP
6 (5)	reflux in IJVs (L)	reflux in IJV (L)
	NP	stenosis: a local CSA reduction of ≥50% across IJV (L)
	NP	block in IJVs (L)
7 (23)	NP	stenosis: a local CSA reduction of ≥50% across IJV (R)
	NP	stenosis: a ≤0.3 cm^2^ at thyroid gland (both sides)
	negative IJVs ΔCSA (R)	negative IJVs ΔCSA (R)

At baseline and at follow-up, for stenosis measurements, method 1 and 2 were used.

Method 1 IJVs stenosis as a local CSA reduction of ≥50% across the IJV. Method 2 IJVs stenosis as a ≤0.3 cm^2^ CSA, measured at the level of the thyroid gland.

ΔCSA  =  CSA_supine_ – CSA_upright_.

Due to lack of assessment, in patient 2, patient 4 and patient 6 criterion V was not assessed at follow-up.

NP not present; this CCSVI criterion was not present.

Abbreviations.

CCSVI, chronic cerebrospinal venous insufficiency; CSA, cross section area; IJV, internal jugular vein; L, left; MS, multiple sclerosis; NP, not present; R, right; VV, vertebral vein.

At follow-up 2 new CCSVI patients with MS were detected. Both patients had one positive criterion at first session (Table 3, patient 6 and patient 7). One patient with a negative ΔCSA at both sessions had 23 weeks after the first session also a new stenosis in the IJVs at both sides at follow-up (Table 3, patient 7). The other patient with reflux in the same IJV at both sessions had at follow-up examination, assessed 5 weeks after the first session, additionally a stenosis and a block in this same IJV (Table 3, patient 6). Despite this patient was not assessed for criterion V at follow-up, he already fulfilled at least 3 of the other 4 criteria.

Besides this variation in presence of CCSVI criteria over time, criterion III (IJV stenosis) shows variations of type of stenosis over time. We used two techniques in our study; a IJVs stenosis as a local CSA reduction of ≥50% across the IJV (method 1), and a CSA of the IJV of ≤0.3 cm^2^ measured at the level of the thyroid gland (method 2). In patients with MS as well as in HC, this criterion switched, during the assessments at baseline and at follow-up, from stenosis defined as method 1 to a stenosis defined as method 2 and vice versa.

## Discussion

The main outcome of our study is that cerebrospinal venous drainage appeared to be dynamic and changes over time, which means that the presence of CCSVI is not constant over time. Without any intervention at follow-up 3 (60%) of the 5 CCSVI-positive MS patients no longer fulfilled the diagnosis of CCSVI, while 2 new MS patients with CCSVI were found at follow-up. Given our results of this natural course of the cerebrovenous outflow system, it appears that this is very dynamic over time, which, to our knowledge, has never been documented before. This seems to be in contradiction with the hypothesis that a congenital origin of venous malformations could play a causative role in MS [1].

Besides this variation in CCSVI criteria over time, the IJV shows physiological variations of its diameter, depending on the subject`s body position, the intrathoracic and central venous pressure [11] and variations of type of stenosis over time. Doepp et al. stated already that the significance of a suspected IJV-stenosis cannot be established solely by measuring the CSA [11]. The subject`s body position, the intrathoracic and central venous pressure are not included in the procedure to detect CCSVI as described by Zamboni et al. [1], [2], [12], [13].

We evaluated the IJVs dynamic variations of the diameter in one HC. In 9 assessments a stenosis (CSA being ≤0.3 cm^2^) was observed in the left IJV, during the other 12 measurements the CSA was normal being ≥0.3 cm^2^.

In our present study we used two different techniques for measuring stenosis in the IJV, both methods mentioned by Zamboni in different studies [1], [2], [12]–[14]; a IJVs stenosis as a local CSA reduction of ≥50% across the IJV (method 1), and a CSA of the IJV of ≤0.3 cm^2^ measured at the level of the thyroid gland (method 2). The type of stenosis sometimes switched, during the assessments at baseline and at follow-up, from stenosis defined as method 1 to a stenosis defined as method 2 and vice versa. These measurements strengthen our main conclusion that cerebrospinal venous drainage is dynamic and changes over time.

Besides these variations, the IJV shows physiological variation per side, the right IJV having a greater CSA in 68% in comparison to the left side [15], [16]. In our recent publicated study the median CSA measured at the level of the thyroid gland at both sides was found to be asymmetric (1.01±0.58 cm^2^ and 0.71±0.32 cm^2^ for the right and left IJV, respectively) and most reductions defined as a CSA ≤0.3 cm^2^ were at the left side (left-side stenosis in 19 patients with MS (52.7%) and 15 HC (62.5%). Both the natural course of the cerebrovenous outflow system as well as the physiological variations of the IJV in diameter over time and per side are not included in the original CCSVI protocol [1], [2], as well as in the new protocol by Zamboni et al. [12].

Our study has some limitations. Although we tried to keep the study blinded for the technicians a possible bias in the patients with severe deficits cannot be excluded, because during the examinations the patients had to change positions. Due to lack of assessment, criterion V was not assessed in all 52 patients with MS and 28 HC at follow-up.

Our findings call strongly into question the validity of using ECD as a proper and reliable test for the diagnosis of CCSVI and to measure venous reflux or stenosis in an attempt to establish the CCSVI criteria. Spontaneous resolution of venous stenosis is a strong argument against using surgical procedures to treat these so-called venous stenosis in MS patients.

This is crucial given the fact that these surgical procedures were increasingly offered to MS patients in the period between 2009 and 2012. Not only is there a great variability in anatomical cerebrovenous outflow routes in MS patients and HC [11], but this study shows that also the (ab) normal ultrasound findings in CCSVI change over time.
